# A Narrative Review of Peer-Led Positive Psychology Interventions: Current Evidence, Potential, and Future Directions

**DOI:** 10.3390/ijerph19138065

**Published:** 2022-06-30

**Authors:** Maike Neuhaus, Tarli Young, Laura J. Ferris, Charlotte L. M. Grimmel, Natasha Reid

**Affiliations:** 1 Centre for Health Services Research, The University of Queensland, Woolloongabba, QLD 4102, Australia; n.reid@uq.edu.au; 2 School of Psychology, The University of Queensland, St Lucia, QLD 4067, Australia; t.young@uq.edu.au; 3 School of Business, The University of Queensland, St Lucia, QLD 4067, Australia; l.ferris@uq.edu.au; 4 Independent Researcher, Port Vila, Vanuatu; hello@charlottegrimmel.com

**Keywords:** positive psychology, positive psychology interventions, flourishing interventions, peer-led positive psychology, complete mental health, peer-led intervention, peer support

## Abstract

Positive psychology interventions are an effective means for cultivating flourishing, addressing low levels of wellbeing, and preventing languishing. Peer-led interventions can be a particularly advantageous delivery method of positive psychology interventions, as participants tend to respond more favourably to people that they can identify with personally. Such interventions have been applied in a variety of settings and populations, but the literature on peer-led positive psychology interventions has not yet been summarised. This paper provides a narrative overview of peer-led positive psychology interventions. We reviewed relevant peer-led interventions, assessed the available evidence on their effectiveness, and highlighted promising opportunities for peer-led positive psychology interventions. We found that the majority of the studies were observational in design but showed a high level of acceptability for participants across the reviewed domains. In particular, schools, workplaces, the aged care sector, and community settings are noted as promising target domains for these interventions. However, more studies—particularly high-quality research—will be needed to comprehensively test the effectiveness of peer-led positive psychology interventions. We discuss opportunities for future research in this field.

## 1. Introduction

Positive psychology is a subdiscipline of psychology that concerns itself with scientifically informed approaches to what makes life worth living, focusing on aspects of the human condition that promote fulfilment, happiness, and flourishing [1]. As such, positive psychology forms a counterpoint to traditional or clinical psychology, which tends to focus on psychopathology and the treatment of mental illness [1]. A central tenet of positive psychology is the acknowledgement that alleviating mental illness does not automatically lead to wellbeing [2]. Consequently, an alternative model of complete mental wellbeing has emerged. The dual continua model of complete mental health conceptualises two separate (albeit correlated) dimensions: one describing degrees of mental illness and one describing the presence of wellbeing or mental health [2]. The latter ranges from languishing (low wellbeing) to flourishing (high wellbeing), and moving people along this continuum has been a key focus of positive psychology.

In accordance with this model, positive psychology interventions (PPIs) are considered a valuable tool for increasing people’s wellbeing. PPIs have been successfully used to increase wellbeing and improve tolerance of distress [3,4,5,6,7,8]; most recently, they have been proposed as a means to address the widespread decreases in global wellbeing associated with the COVID-19 pandemic [9,10]. PPIs can be delivered in a range of formats, including self-led, facilitated, or via e-platforms. However, an increasingly promising approach to delivering wellbeing interventions is through peer facilitators.

Definitions and operationalisations of ‘peer-facilitators’ or ‘peer-leaders’ vary greatly in the literature, where peers are understood as individuals sharing certain demographics with the target group [11] or who have experiential knowledge of an outcome addressed by a program—a definition most commonly used in mental health research [12]. Importantly, peer leaders can play a special role in extending or complementing professional health and wellbeing services, with research showing that target groups tend to respond more favourably to interventions delivered by peers [13]. This aligns with research showing that a key predictor of successful behaviour change is social support [14].

While some PPIs already integrate a peer-led approach, the literature has yet to be summarised. We see the potential to amplify the uptake and impact of PPIs through more widespread understanding and adoption of peer-led methods and approaches. In addition to this, summarising the literature in this area is key to enabling science-based use and uptake of peer-led methods. Thus, the purpose of this paper is to provide an overview of peer-led positive psychology interventions. As this literature has never been summarised and the field is nascent, we conducted a narrative review as a starting point to generate further discussion. The aims of this review are three-fold:

(1). To provide an overview of peer-led PPIs in various settings and their findings;
(2). To identify promising opportunities for peer-led PPIs in several domains;
(3). To make recommendations for future research on peer-led PPIs.

In the following section, we review the evidence regarding peer-led PPIs, their impacts, contexts, and relevant populations. Studies were identified via literature searches and included whether they reported outcomes of a positive psychology intervention facilitated by peers; we excluded studies not published in English. For the purpose of this review, we defined ‘peer-led interventions’ as programs facilitated by non-expert individuals who may have received training in the subject matter and, if so, for the sole purpose of delivering the program. The notion of ‘peers’ can include the alignment of demographic variables such as age group and living location, but it can also include the alignment of shared experiences, such as shared mental health challenges or career identities. While a systematic literature analysis was outside the scope of this research, we searched the PubMed database for relevant literature. Furthermore, we applied three groups of search terms pertaining to ‘peer-led’, ‘positive psychology’, and ‘interventions’. Search results were further complemented with studies known to the authors, as well as through additional relevant papers identified through reference lists. Studies were considered relevant if they were facilitated by peers and included an intervention targeting mental wellbeing, in line with the dual continua model described above. We begin the review with a brief overview of positive psychology interventions.

## 2. Positive Psychology Interventions: Purpose, Examples, and Evidence

Positive psychology interventions are designed to promote wellbeing and other positive variables within individuals and groups, thus boosting overall wellbeing and providing support to cope with negative experiences [3,4]. PPIs include an extensive range of activities, such as acts of kindness, writing about meaningful moments, building optimism, gratitude writing, savouring positive emotions, and acting in line with one’s character strengths [3]. They are often undertaken online, in classrooms, through books, or within therapeutic settings, thus making them a helpful tool for therapists, teachers, coaches, and individuals seeking to improve or maintain wellbeing and flourishing [3].

The term ‘PPI’ is not easily defined, due to the diversity of contexts and methods used to invoke positive change. Parks and Biswas-Diener [15] proposed three specific inclusion parameters. Firstly, PPIs should primarily aim to increase a positive variable (e.g., subjective well-being, positive emotion, and meaning), rather than reduce a negative variable. Secondly, PPIs ought to be evidence-based—that is, empirical evidence must demonstrate that the intervention successfully changes the target variable (e.g., research must show that a wellbeing intervention actually increases wellbeing). Lastly, PPIs should be appropriately tailored to the population and must demonstrate positive outcomes for the specific population receiving the PPI. For instance, a gratitude intervention may be helpful for university students but detrimental for recent trauma survivors [15].

There is mounting evidence that PPIs are efficacious and adaptable. A recent meta-analysis of 419 RCTs of psychological interventions to improve mental wellbeing showed that single- and multi-component PPIs were impactful in clinical and non-clinical populations [16]. In particular, mindfulness-based interventions showed strong efficacy, as well as those PPIs in multi-component format for multiple population types, i.e., people with a mental illness, people who were physically unwell, and the general population. However, combining several intervention components such as these can make it difficult to determine the effectiveness of individual components. Furthermore, PPIS that were based on behavioural or acceptance and commitment therapy, as well as reminiscence interventions, were particularly effective [16]. Unlike traditional psychological interventions that target only clinical populations, PPIs can be effectively employed across various populations and have been used in education [17], organisations [18], clinical populations [4,5], healthy populations [19], local communities [20], and at-risk groups [21]. Thus, a key strength of PPIs is their breadth and flexibility. Below, we discuss three quintessential PPIs as examples—gratitude, strengths, and kindness.

Gratitude PPIs aim to build feelings of gratitude by encouraging participants to reflect on things for which they are grateful [22]. This can involve expressing gratitude to other people, as seen in studies of ‘Gratitude Letters’, where participants write their thanks to another person [23]. This exercise promotes increased positive effects, life satisfaction, meaning in life, and decreased negative emotions [24,25]. Other gratitude interventions are more self-reflective tasks, such as gratitude journaling, which leads to benefits such as improved physical health [26], meaning [27], engagement [27], life satisfaction, and positive affect [28]. The most common gratitude intervention is named ‘Three Good Things’ and involves participants writing or thinking about three good things that happened over the last day and reflecting on why those things may have occurred [29]. This activity has been shown to lead to increased happiness [30], as well as decreased depression and emotional exhaustion [31].

While gratitude focuses on sources of positivity in life, strengths-based PPIs aim to increase peoples’ awareness and use of their character strengths [15]. These interventions often involve a strengths assessment (e.g., the Values in Action character strengths test [32]) and ask participants to use their identified strengths in a new way or develop these strengths [29]. These interventions can lead to increased happiness and decreased depressive symptoms [29], while also improving life satisfaction [33] and happiness [30]. Importantly, some researchers have emphasised the need to conceptualise strengths as an evolving part of a person, rather than a fixed factor [34]. Others have theorised that strengths-based PPIs may be best delivered while encouraging participants’ ‘practical wisdom’ [35], i.e., the ability to use one’s strengths when appropriate and beneficial.

Kindness PPIs take a more interpersonal approach than strength-based PPIs and build on research, indicating that happiness and kindness have a mutually supportive function [36]. The most common PPI in this category is called ‘Acts of Kindness’ and entails participants undertaking a series of kind acts for other people. The acts can vary widely—from buying a cup of coffee, opening a door for someone, or providing aid for someone in need. This intervention has been shown to lead to increased wellbeing and happiness [37] and is particularly effective when multiple acts are performed in one day [38]. Kindness PPIs have the added benefit of promoting interpersonal wellbeing [39] which is less often the focus of PPIs [34].

## 3. The Role of Peer and Social Support in PPIs

Above, we have described the effects of three seminal categories of PPIs, although it should be noted that PPIs touch on many other areas, such as mindfulness, community activities, self-compassion, etc. Next, we consider the rationale for including peer-based components within PPIs. A key factor that has been found to amplify the impact of PPIs is social support. For example, students who complete an optimism intervention and read an empathetic peer testimonial had larger increases in positive effects than other participant groups without the peer testimonial [40]. Similarly, participants undertaking acts of kindness, who read a supportive message from a peer, experienced greater improvements in happiness than participants who did not receive social support [41]. Both these examples demonstrate that peer support can increase the impact of PPIs, even without face-to-face contact. It is also possible to target social support through PPIs. For example, Appiah and colleagues [20] developed a PPI program for adults in rural Ghana that aimed to build peer support, psychosocial skills, and collaborative therapeutic relationships. Participants of the intervention reported an increased sense of positivity and well-being, along with stronger social networks and relationships. These studies demonstrate that adding peer components to PPIs can increase their impact. This may be due to a higher likelihood of participants relating to a peer leader and, by means of social learning, cultivating stronger self-efficacy, in order to realise the targeted change themselves [42]. Moreover, deeper consideration of peer-led PPIs provides a chance to respond to concerns within the field regarding the overly individualistic focus of many PPIs [34] and need for interventions that are more interpersonal and will affect group-level outcomes, such as friendship, trust, and connectedness [43]. In the following, we summarise existing literature reporting on peer-led PPIs and offer recommendations for different settings and populations.

## 4. Implementation of Peer-Led PPIs across Different Settings

The use of peer-led approaches with PPIs has been slowly growing. Over the last decade, a number of peer-led PPIs have been implemented and evaluated in countries such as the United Kingdom [44,45], New Zealand [46], and the United States of America [47,48,49,50] and administered across domains such as educational institutions [46,47,48,49], healthcare settings [45,47], and the wider community [44,49]. Participants included adolescents [48] and adults [44,45,46,47,49,50]. Within these studies, peer support has been characterised in various ways. They included ‘champions’ or elected peer leaders, i.e., people who are part of the same workplace or student cohort volunteering their time to support others [46,47,49,50], people with lived experience in the intervention target outcome [45,48], or a group of people with shared characteristics, which receives support facilitation from a non-professional [44]. Peer-led PPIs also varied in their duration, ranging from a few weeks [44,45,48] to a year [50].

Past research indicates that peer-led PPIs have led to a broad range of positive outcomes. These include increased resilience [44,46,47,48], happiness [47], optimism [49], forgiveness [49], spirituality [49], gratitude [45], mindfulness [45], positivity [45], empathy [50], hope [45], life satisfaction [48], personal recovery [48], mental health [46], and self-efficacy [50]. In terms of secondary outcomes, studies also report reductions in stress and burnout [47,49], depressive symptoms [46,48], and anxiety [46,49]. However, the methodological quality across studies to date is relatively low, with most studies having employed a quasi-experimental design [44,45,47,48,49]. This is discussed in further detail below. An overview of the studies included in this review is provided in Table 1.

In the following sections, we focus on specific domains—discussing the existing literature and specific opportunities for the implementation of peer-led PPIs across educational institutions, community settings, workplaces, and the aged care sector.

### 4.1. Peer-Led PPIs in Universities and Schools

Positive psychology interventions are an effective means of enhancing student wellbeing, social relationships, and academic performance [53,54], and their use is on the rise, especially in the USA and Australia [55]. In the university setting, a number of such studies have been conducted, targeting medical students and residents. For example, Aggarwal and colleagues undertook a qualitative study examining whether wellness curriculum incorporating strategies from positive psychology could be easily and effectively implemented [47]. A total of 188 medical residents participated in the initial session. The subsequent 11 weekly sessions were led by medical residents who had received a short training by the faculty advisor, along with a wellness handbook. During weekly sessions, peers guided the residents through 2–3 exercises from the handbook, including exercises such as five good things, reframing, and diaphragmatic breathing. At the end of the intervention, qualitative feedback was collected, in which participants stated that the sessions were effective. Residents in four participating departments chose to continue the weekly sessions on a voluntary basis.

In a similar study, Moir and colleagues studied the feasibility and effectiveness of a peer-led, mindfulness-based program with 232 medical students in New Zealand [46]. Peer leaders were 12 medical students that had been selected via an application and anonymous voting process. Peer leaders received small-group and mindfulness training over eight weeks. The mindfulness intervention involved weekly mindfulness sessions run by the peer leaders, and participants were encouraged to approach peer leaders for face-to-face support. Participants showed improvements in depression and anxiety scores, as well as quality of life and resilience. Results were not significantly different for the control group. However, while participants of the control group were not explicitly invited to the training, they were not excluded from intervention components—a methodological choice that may have contributed to the lack of difference in outcomes between the groups.

Interestingly, one study explored the impact of a peer support program on peer supporters themselves in 38 medical students in the United States [50]. Peer supporters were taught by a psychologist to promote positive mental health and reduce stigma, and they were subsequently provided walk-in sessions and bi-annual outreach events throughout the academic year. Training for peer supporters included active listening, motivational interviewing, and mindfulness/guided relaxation strategies. On average, peers delivered four support sessions during the academic year. Measures taken at the start and end of the academic year showed peer supporters had increased empathy and enhanced self-efficacy in helping others as a result of participation in the program. This indicates that peer-led PPIs in university settings can be helpful for the peer leaders, in addition to participants.

Together, these studies demonstrate that it can be feasible to integrate peer-led PPIs into university curricula [47]; they can improve some important wellbeing outcomes [44] and benefit both the target audience and peer leaders [50]. However, more research is needed that is of stronger methodological quality and explores a broader variety of positive psychology outcomes.

In contrast to the literature on peer-led PPIs in universities, there is very little published research on peer-led PPIs in a school setting. In one study, Vela and colleagues examined the efficacy of a seven-week high school-based intervention, in order to increase resilience and personal recovery and reduce depressive symptoms in 67 Latina/o adolescents in grades 9 and 10 [48]. Participants were selected based on having reported mental health needs to the school counsellor. The intervention facilitator (an adult) was defined as a peer on the basis that they also had lived experience with personal struggles of mental illness, which they shared with the group. The intervention involved seven sessions that aimed to support adolescents to express their emotions, identify gratitude, and develop hope for the future. Participants in the intervention group obtained higher scores on both resilience and personal recovery attitudes, as well as lower scores on depressive symptoms, from pre- to post-intervention.

A qualitative study by Chung, Monday, and Perry [51] examined young people’s perceptions of a peer-led, drama-based workshop program to promote adolescent wellbeing. High school students facilitated a series of workshops on youth-relevant topics, with peer recipients who were urban students in elementary and middle schools (*n* = 4733). The study examined peer recipients’ perceptions of workshop content and implementation. Findings suggest that the workshops helped the peer recipients to learn more about wellbeing issues that were important to them, but perceptions varied by age. This study offers useful insights for the designers of peer-led PPIs, in terms of factors to consider in crafting material to best match students’ developmental needs; drama or arts may be a helpful format for delivery of peer-led PPIs in future. However, the study neither gathered quantitative data on the program’s direct effect on wellbeing nor made any comparisons with a control group.

Beyond the previous studies, school-based PPIs reported in the literature were largely delivered by teachers, researchers, or trained psychology specialists [54]. The relative absence of peer-led PPIs in schools is at odds with the broader pedagogical acceptance of peer-led interventions in other areas of school life, where the value of peer-led mentoring, helping, tutoring, and mediation have been successfully integrated and generally show benefits for peer recipients and leaders [56]. There may be perceived regulatory and practical barriers for implementing peer-led PPIs in school settings. Previous studies have found that funding and time pressures in educational settings have made it difficult for schools to take up new initiatives, such as PPIs, in the past [55]. Furthermore, it may be economically challenging to integrate PPIs into the school system, instead of targeting individual or selected classrooms [53]. Targeted or integrated solutions require some level of PPI training for teachers, and it is worth considering whether selected students can be included in the training, especially at the secondary school level. As seen in the study by Vela and colleagues, PPIs facilitated by adolescent peers show promise for supporting students in undertaking practices cultivating resilience and personal recovery, as well as reducing depressive symptoms [48]; however, further research is needed.

### 4.2. Peer-Led PPIs in Community Settings

While no consensus exists regarding the definition of ‘community’, it can be characterised as a group of people “who share distinctive characteristics associated with common interests or identities” [57]. In the past, PPIs have been implemented with diverse community populations, including elderly people, people with a physical health condition, people with low socio-economic status, families, at-risk groups, unpaid carers of dependent people, and churchgoers [57]. They have been facilitated in person, over the phone, and online. Interestingly, most of the community PPIs to date have focused on enhancing the wellbeing of individuals, rather than the collective group, a phenomenon of positive psychology research that is commonly criticised [58]. However, the majority of these studies tend to have a beneficial impact on individuals. The articles included in this review have targeted a group of unemployed men [44], marginalised men of low socio-economic status [49], a group of cancer survivors [45], and veterans [52].

Robinson and colleagues explored the effect of a community mental health intervention with a facilitated peer support for unemployed men, aged 45 to 60, in England [44]. Peer leaders were identified as members of the group and did not receive specific training before participating in the group activities. The intervention was based on Mind’s resilience program and used practical group-based activities, such as gardening, crafts, and refurbishing, in order to build social networks and develop positive psychological coping strategies. The average program length was eight weeks. Through qualitative interviews conducted before and after the program, participants reported increased resilience and improved trust in informal social connections.

In another community-based study, Ali and colleagues examined the effects of a 3-month peer-led remote intervention on mental health and spiritual wellbeing in a sample of 79 Black participants living in the Bronx, New York [49]. Participants were selected via convenience sampling from congregations of diverse denominations. Peer leaders were chosen by religious leaders and received approximately eight hours of training across four sessions. The intervention involved eight online group sessions that focused on mental health problem identification, improving overall spiritual well-being, and addressing feelings of anxiety, stress, and trauma through spirituality-based strategies. In addition, the curriculum incorporated cognitive-behavioural approaches to changing negative thinking patterns and developing optimism. Post-intervention results indicate that participants had higher scores on sense of community, social support, and flourishing, as well as significantly lower depressive symptoms. In qualitative interviews, participants also reported a stronger sense of control, better communication with others, greater emotional balance, and increased self-awareness, as well as decreased feelings of sadness, loneliness, and anxiety.

In a healthcare setting, Martin and colleagues examined the feasibility, adherence, and effects of a 6-week digital peer-facilitated intervention on well-being in 114 cancer survivors at the end of treatment/surgery [45]. Peer facilitators had also been affected by cancer in some way. The program was delivered weekly in groups of up to 20 participants, focusing on goal setting, gratitude, mindfulness, positive emotions, and hope. Peers delivered support via social networks and interactive activities. Compared to pre-intervention levels, participants obtained higher scores in positive mental well-being, hope, and gratitude assessments. In addition, participants reported decreases in scores for depression, anxiety, cancer-related fatigue, and fear of recurrence after the program.

Finally, in one unpublished mixed methods study [52], a community-based peer-led initiative with veterans and emergency first responders was described. The intervention aimed to teach veterans mind–body self-care skills (psychoeducation and mindfulness meditation) over a 10-week program. The program material was delivered by trained veteran facilitators. Qualitative findings indicate that peer involvement was experienced as a positive, supporting participants to feel hope (‘they could do it, I could do it’) and facilitators to feel valued (‘it’s rewarding seeing the reactions of the people, to see them become open’, p 54). Feeling understood was also a theme—‘we could relate to each other’ (p 74). However, quantitative findings were not reported.

In line with these studies, other published works recognise the promise of peer-led PPIs in community settings, such as peer coaching in the delivery of youth services [59], peer-led community health workshops in refugee communities [60], or peer-led resilience and capacity building in urban and regional communities [61]. However, these studies tend to lack empirical data specifically establishing the intervention effectiveness or efficacy within the setting. In one example, the ‘Wheel of Wellness’ (WoW) framework was implemented in three ‘Wellbeing Hubs’ across Queensland, Australia. WoW is a flexible, community-based mental health program underpinned by positive psychology, that conceptualises wellbeing as comprised: (1) body, (2) mind, (3) spirit, (4) people, (5) place, and (6) planet. It was originally implemented as part of a UK program called Well London, which a cluster-randomised trial found to be effective in delivering health, wellbeing, and social benefits directly to participants, but not their communities, more broadly [62]. Moreover, a nested qualitative study into Well London found the extent of benefits was tempered by the physical and social characteristics of each community [63]. In the iteration conducted in the state of Queensland, peer community members were offered training, along with mental health service providers and community service workers. While WoW was implemented in the Queensland communities for a period of some years, with generally positive feedback from its participants, only one peer-reviewed paper examined its local implementation, and none have reported on its longer-term outcomes or impacts [61]. This highlights the need to prioritise peer-reviewed evaluation of programs across diverse communities and settings, so that learnings can be assessed, shared, and utilised.

Importantly, there may be groups in communities for whom peer-led PPIs are not considered feasible or acceptable by the participants themselves. One study by Bassett and colleagues [64] implemented a PPI with a group of Black women living with HIV, with the aim of promoting positive affect, wellbeing, and gender empowerment. These researchers specifically asked participants whether they felt the program should be peer-led, and found that participants did not feel that a peer-led approach would be suitable for them:

*When asked if this group should be peer-led, several participants gave a resounding no, one shook her head vigorously against the idea, and one participant noted that it would be difficult for another WLWH [woman living with HIV] to lead the group, saying, “It would be touchy to have someone switch from peer to staff and back.” Participants explained that they want to have this group led by someone with content expertise and who can keep them on task with respect to learning new skills. (p. 1744)*

This approach models a quick, direct, and participatory way to empower participant-driven design and better understand participants’ receptiveness, hesitancy, or opposition to the concept of peer-led interventions in particular settings. Broadly implementing this approach could facilitate peer-led PPIs, where they are most likely to succeed.

### 4.3. Peer-Led PPIs in Workplaces 

Workplaces are another potential setting in which to offer peer-led PPIs. Employees spend a significant portion of their day at work, resulting in more opportunities to engage with peers, and there are both personal and workplace-related incentives, such as improved retention, reduced sick leave, and better performance [63].

Despite this, there is a dearth of research on peer-led PPIs in the workplace [65]. A meta-analysis by Donaldson et al. (2019) on general positive psychology interventions in the workplace found that PPIs had a small positive effect on improving desired workplace outcomes (such as job performance, job well-being, satisfaction, etc.), as well as a small to moderate effect on reducing undesired workplace outcomes (such as negative affect, job stress, emotional exhaustion, and negative performance) [66]. However, there appears to be little reference, specifically, to PPIs led or championed by peers within these workplace interventions. It is noteworthy that the meta-analysis by Donaldson et al. (2019) suggests that workplace-based PPIs are more effective when delivered in group settings [66]. This is in contrast with previous reviews and meta-analyses that found that individually targeted interventions were more effective [5,7]. It may be that these group-based interventions facilitate relationships and collaboration in workplaces, potentially increasing workers’ self-identification with their workplace [67]; however, further research is required to resolve the inconsistency and determine whether workplace identification is the psychological mechanism of action.

A limitation of the literature to date is that most PPIs in workplace (and other) settings focus on individual-level traits, neglecting the broader socio-environmental influences on behaviour, mood, and wellbeing [68]. This is especially important in workplaces where group or team-level states, traits, and behaviours co-exist with individuals and bi-directionally influence one another [69,70]. Of note is the finding that longer-term structural changes are more likely to be more effective for sustained improvement [68]. Therefore, an area of significant opportunity in the field is the trial and evaluation of peer-led PPIs in the workplace. The literature on ‘peer champions’ at work for health and well-being promotion more broadly is well-established, showing their critical role in establishing motivation for change and assisting in program uptake [71]. Champions have been defined as “employees who are not necessarily experts in the field of health and wellness, but have a passion for it, personally and professionally, in the sense they want to promote wellness among their colleagues.” [56] (p. 59). While a range of workplace wellness programs exist, including those with peer ‘coaches’ or ‘champions’, the vast majority of these tend not to have been rigorously evaluated [72]. For instance, a peer support program for anaesthetists [73] provides a peer-led crisis response mechanism for anaesthetists to receive outreach from trained peers after an adverse event, but also aims to establish a culture of wellness and proactive self-care in the workplace, as led by peer anaesthetists. The program shows uptake and engagement by workers. However, the program was not evaluated, in terms of its effects on wellbeing. This is consistent with a broader movement in peer support for high-pressure job roles—not only anaesthetists [74], but other professions such as first responders, military [75], and people in construction (see, for example, ‘Mates in Construction’ [76]). There have been calls for a stronger evidence base and more rigorous evaluation studies, in order to better understand the effect of promising peer-led peer support initiatives [77]. New studies and programs are needed to investigate peer-led PPIs in organisational settings.

### 4.4. Peer-Led PPIs for Older Adults and Aged Care Settings

Ageing can lead to functional and cognitive impairments that may make older adults more prone to depression and anxiety, poor life satisfaction, and languishing [78]. “Successful ageing”, a term originally coined by Carol Ryff three decades ago [79], includes several constructs that are closely related to measures of positive psychology, including self-acceptance, positive relations with others, autonomy, environmental mastery, life purpose, and personal growth [80]. Therefore, PPIs may be a useful tool in this segment of the population. However, there is a lack of published studies on peer-led PPIs for older adults or in aged care settings, thus indicating a need for more research in this area. 

Systematic reviews have shown that PPIs led by professionals are effective at improving wellbeing in older adults, including measures of life satisfaction, resilience, positive affect, and happiness [81]. These studies have also shown that interventions can reduce symptoms of depression, anxiety, and stress [81]. The most utilised type of PPI for older adults was reminiscence interventions, which use past experiences to support psychological wellbeing. Similarly, peer-led programs, which mostly focus on physical activity, general wellbeing, and/or nutrition, have been shown to improve a vast range of health and wellbeing measures in older adults in the community [82], as well as those in retirement living [83].

Importantly, there may be unique strengths and needs within this population that differ from younger populations. These may include strengths such as greater life experience, more leisure time, or higher levels of wisdom, as well as issues associated with ageing, such as reduced physical capacity, loss of a spouse and other grief experiences, reduced sense of purpose or loss of group memberships associated with transitioning away from work, loneliness, and social isolation. Evidence-based strategies to address changes through ageing, such as “rational emotive behaviour therapy (REBT)”, can help people identify irrational beliefs and thought patterns that lead to negative effects and behaviours [84]. While books and other resources for individuals to learn these techniques have existed for decades [85], peer-led approaches to implementing these interventions have yet to be tested and are, thus, an area for future research.

## 5. Summary and Future Directions

This paper provided the first narrative review of peer-led positive psychology interventions. It highlighted a small number of research studies describing and demonstrating the feasibility and effectiveness of peer-led PPIs, particularly in university and community settings. However, research on peer-led PPIs remains in its infancy; presently, the quality of studies overall is low. While the reviewed literature provides a theoretical rationale for the exploration of peer-led PPIs in schools, workplaces, and aged care settings, more high-quality empirical evidence is needed.

Based on the current evidence-base, we propose the following suggestions for future research in this field. Firstly, given the prevalence of quasi-experimental studies in this area, future research would benefit from a greater utilisation of experimental designs, especially RCTs, and longer follow up periods to examine whether the positive impacts observed are sustained over time. A particularly useful line of investigation would be for more studies to directly compare the impact of peer support versus non-peer support facilitation, in the context of PPIs. This methodological approach was adopted by Spence and Grant, in the context of a life-coaching intervention [86]. Interestingly, these authors found that life coaching delivered by professional coaches, rather than peers, had a more beneficial impact on participants’ goal striving. This highlights the importance of rigorous comparison to isolate the operative ‘ingredients’ of peer-led PPIs, as well as to interrogate this across populations and contexts. Notably, there were very few studies from non-Western contexts. This reflects broader observations made of the field as a whole [87] but may be an artefact of our inclusion criteria, such that only studies that were published in English were considered. In addition to the suggestion of examining the relative benefit of engaging peer vs. expert facilitators on primary study outcomes, we further recommend studying the cost implications of peer-led interventions. It would also be helpful to explore when peer-led modalities are not appropriate, such as when extensive training is needed to run interventions effectively.

Furthermore, as mentioned earlier, definitions and operationalisations of ‘peer-led’ or ‘peer-facilitated’ vary greatly in the literature. Ideally, the field ought to reach a consensus regarding the definition of peer-led interventions. At a bare minimum, it would be desirable to define how closely peers need to resemble the target population (e.g., is it sufficient to share lived experience of an outcome of interest or should they share certain demographics?). In the meantime, we recommend that all published studies include an explicit description of their use of the term. Finally, it might be of benefit to explore the benefits of peer-led PPIs across other settings, such as correctional facilities or hospitals. More research is also needed, with regard to the acceptance of peer leaders across diverse community populations. Despite these current gaps in the literature and opportunities for future research, peer-led PPIs are growing in reach and represent a new opportunity for PPI uptake and impact.

## 6. Review Limitations

While this review offers an overview of peer-led PPIs, there are also some limitations to the approach used. The review is based on narrative analysis, meaning that the total corpus of studies may not have been estimated and effect sizes were not meta-analysed. However, this approach is appropriate, given that this literature is nascent and dominated by quasi-experimental designs. With this review, we have provided a useful first step—as the quality and quantity of primary studies on peer-led PPIs grow, we encourage more systematic review methodologies to be deployed, such as scoping reviews and meta-analyses in this area. Another limitation is that this review considered studies published in English only, and future reviews could widen the scope of studies examined to include other languages.

## 7. Conclusions

This narrative review brings together studies on peer-led PPIs for the first time and shows that this is a promising area of research. Most of the studies discussed showed positive outcomes and a high level of acceptability for participants across many domains. There are many methodological issues noted in this field, and greater research is needed to fully test the effects of peer-led PPIs—this approach will not be appropriate to every situation and more research is needed to identify when and how peer-led approaches can best be utilised with PPIs. By bringing the existing research together, the current review offers potential avenues for further exploration in this promising field.

## Figures and Tables

**Table 1:** Overview of relevant studies included in the review.

Study	Country	Setting	Peer Leader (s)	Sample	Study Design	Intervention Duration
Aggarwal et al. (2017) [47]	USA	Hospital	Volunteer medical residents	188 Volunteer residents	Pre-post quasi-experimental	12 weeks
Moir et al. (2016) [46]	New Zealand	University	Elected peer medical students	232 Elected medical students	Pre-post experimental (however, control group participants could attend intervention sessions, as well)	6 months
Abrams et al. (2022) [50]	USA	University	Volunteer students	38 Medical students	Pre-post quasi-experimental	12 months
Vela et al. (2019) [48]	USA	School	Adult sharing lived experience	67 Latina/o adolescents (attending grades 9 and 10)	Pre-post quasi-experimental	7 weeks
Chung et al. (2017) [51]	USA	School	High school students recruited from the same communities	4733 School students aged 6–22 (grades 1–12)	Pre-post quasi-experimental	<1 day
Robinson et al. (2015) [44]	UK	Community	Non-peer professional enabling mutual peer support	53 Unemployed men aged 45–60 years	Pre-post quasi-experimental	8 weeks on average
Ali et al. (2021) [49]	USA	Community	Elected by leaders of the congregations, based on level of involvement in church activities, demonstrated leadership, and interest in becoming a peer educator	79 Black residents (members of congregations in the Bronx)	Pre-post quasi-experimental	3 months
Martin et al. (2020) [45]	UK	Community	Two trained peer facilitators who were “affected by cancer in some way”	114 Cancer survivors	Pre-post quasi-experimental	6 weeks
Clifford (2016) [52]	USA	Community	Trained veteran facilitators	15 Veterans	Pre-post quasi-experimental	10 weeks

## Data Availability

Not applicable.
